# The Roles of Work-Life Conflict and Gender in the Relationship between Workplace Bullying and Personal Burnout. A Study on Italian School Principals

**DOI:** 10.3390/ijerph17238745

**Published:** 2020-11-25

**Authors:** Ilaria Buonomo, Caterina Fiorilli, Luciano Romano, Paula Benevene

**Affiliations:** Department of Human Sciences, LUMSA University, 00193 Rome, Italy; fiorilli@lumsa.it (C.F.); l.romano@lumsa.it (L.R.); benevene@lumsa.it (P.B.)

**Keywords:** psychosocial risks, workplace bullying, gender differences, work-life conflict, school principals, educational organizations

## Abstract

The present study sought to investigate the associations between workplace bullying and personal burnout both directly and indirectly via work-life conflict. Furthermore, the moderating role of gender in these relations was examined. Traditional research on stress at work focuses on the role of dimensions related to job tasks, demands, and organizational support in influencing the risks for stress-related problems in employees. At the same time, other experiences at work may reduce employees’ well-being, such as workplace bullying and family life. Specifically, considering the detrimental role of work-life conflict, it is possible to hypothesize that it would exacerbate workplace bullying’s harmful effects on employees’ health. Moreover, since previous studies have reported mixed or inconsistent results when considering gender differences with the above-mentioned dimensions, it seems worth investigating the role of employee gender in representing (and response to) the bullying experiences. Building on these considerations, this work verifies whether: (1) work-life conflict mediates the relationship between workplace bullying and burnout; (2) gender moderates all the possible relationships among the constructs. Such hypotheses are verified on a sample of school principals, in light of their peculiar job role. Overall, our findings showed that: (1) Workplace bullying and burnout are associated, both with and without the perception of a concurring work-life conflict; (2) Gender does not moderate all the possible relationships among workplace bullying, work-life conflict and burnout. Overall, being female heightens the risk to perceive work-life conflict in general, as well as to be burnt out, when bullied, with and without the presence of work-life conflict; being male heightens the risk to perceive work-life conflict when bullied. Furthermore, the current findings suggest that family demands may influence school principals’ feelings of exhaustion regardless of gender. These findings confirm and expand previous literature, especially concerning a less studied occupation, namely school principals, shedding a new light on their work experiences. Furthermore, the present study offers interesting implications for trainings on principal’s skills and professional identity.

## 1. Introduction

Research on workplace stress and burnout traditionally considers the role of job demands, workplace support, employee perceptions about role clarity and their sense of autonomy and control [1,2,3,4,5]. Although such works are highly informative to describe the processes leading to stress-related health risks, several other experiences, despite less common, could have a crucial role in influencing workers’ levels of stress. Among these, workplace bullying was shown to cover an important role. 

Workplace bullying draws the attention of both researchers and practitioners because of its implications for both employee health [6,7,8,9,10,11] and organizational performance and external image [8,12,13]. As reported by Bartlett and Bartlett, workplace bullying is represented by aggressive behaviors of mild to high intensity, repeatedly directed towards a target [9]. Consistently, it is considered a counterproductive behavior, namely a harmful behavior occurring at work [14]. In the last decades, most organizational research focused on the influence of such recurring episodes on employee performance and organizational outcomes [12,13,15]. More recently, an increasing number of studies underlined the role of bullying episodes on employees’ private life, opening a debate on the effects of counterproductive behaviors beyond the organizations (e.g., [1,10]). Building on this literature, work-life conflict or work-life interference seems to represent effectively one of the processes by which bullying could affect life outside the workplace [6,15,16]. Previous studies, indeed, have shown that the negative effects of workplace bullying on employees’ occupational health and quality of life goes through higher levels of work-life conflicts [17]. 

Mental health is among the most tackled effects of workplace bullying in literature considering employee private life outcomes [11]. This construct has been measured through anxiety, depression, stress, and burnout scales [11]. Burnout is usually considered as a state of physical and emotional exhaustion, depersonalization and low sense of accomplishment, related to the employee work life [18]. At the same time, other authors stated that burnout, intended as physical and emotional exhaustion [19], may occur in several different life contexts [20]. These considerations led to a distinction among personal burnout, namely “the degree of physical and psychological fatigue and exhaustion experienced by the person” [14], (p. 197), work-related burnout, namely “the degree of physical and psychological fatigue and exhaustion that is perceived by the person as related to his/her work” [14], (p. 197), and client-related burnout, namely “the degree of physical and psychological fatigue and exhaustion that is perceived by the person as related to his/her work with clients” [14], (p. 197). While the least two dimensions are more attuned to the attribution of symptoms to the workplace (either in general or regarding clients), the personal burnout dimension addresses the health-related negative outcome, as it is generally perceived by the individual. Taking into account this perspective, it seems that personal burnout represents an effective construct when considering the potential broad negative effects of workplace bullying on employee life. 

Current literature on workplace bullying, work-life conflict, and burnout tackles several occupations, by frequently addressing helping professionals, such as physicians, nurses, psychologists, and teachers [21]. This work focuses on helping professionals in a leading position in schools, namely school principals. According to reports built on OECD data [22,23] and further literature [24,25,26,27,28], school principals encompass two functions. Firstly, they are school leaders and administrators: they organize and implement the administrative procedures, deal with bureaucracy, define school relationships with external stakeholders, and manage the school staff. Secondly, they are care workers: they are entitled to define the school rules about discipline, deal with student misbehaviors, and mediate the relationships among schools, teachers, and families. This double role makes principals’ work experience unique, even in terms of work-related relationships. Some studies report principals describing their leadership role as emotion- and relation-based [29,30]. At the same time, in a recent study, school leaders were reported as less likely to meet teachers and other school-workers “by chance” and have unplanned, informal interactions with them [31]. 

Overall, this work is intended to address the associations between workplace bullying and personal burnout, as mediated by work-life conflict. This general aim is further defined by two elements: a moderating variable, namely gender, and a specific context and job role, namely educational organizations, and their leaders. The following paragraphs will detail the work hypotheses. 

### 1.1. Workplace Bullying and Gender Differences 

Workplace bullying involves persistent aggressive behaviors towards an employee [14,32,33], who perceives such conducts as abusing and difficult to defend himself/herself from [12]. Bullying behaviors may regard the employee work or individuality [9]. In the first case, namely work-related bullying, an employee workload, process or evaluation is manipulated, in order to penalize him/her. In the second case, namely personal bullying, the employee is subject to gossip, isolation, personal jokes, or, in more severe forms, threats. The consequences of such behaviors regard not only organizations (e.g., in terms of productivity, legal issues and reputation [9,34,35]), but, above all, employee health [9,34,35,36,37]. In a meta-analysis of 70 studies, Verkuil and colleagues [11] showed that workplace bullying is associated to stress, depression and anxiety, both in cross-sectional and longitudinal research. Workplace bullying is deemed a multicausal phenomenon [38], associated to personal, organizational, and cultural dimensions [39]. Among these, in a recent literature review [39], gender was reported as a significant predictor of the involvement in bullying episodes at work, with women facing a higher risk of being bullied than men. This datum is recurrent in different occupations [40,41,42], and regards both employees and managers [43]. To the best of our knowledge, few studies have addressed the role of gender in workplace bullying episodes towards workers in leading positions. Among these, it is particularly relevant to consider that female managers may be more at risk for bullying because of dominant sexist attitudes in the organizational sectors [43,44]. At the same time, it must be noted that a recent review a few studies showed neither gender effect [45,46], nor a higher risk for males [47,48]. With specific reference to school principals’ work experience, the organizational social climate as well as the job position seem to be valuable antecedents to be taken into consideration. In the first case, as widely reported in workplace bullying research, such behaviors stem from social interactions and the social climate within the organization [8,38,49,50], and considered the isolated position of principals within the school hierarchy [51,52,53,54], this aspect could act as a risk factor. In the second case, several studies on supervisory workplace bullying showed that workers with management roles are more exposed to workplace bullying than workers in the same organization with different job positions [55,56,57,58]. Thus, it is likely that principals’ peculiar role at school could heighten the risk of being exposed to bullying conducts. 

### 1.2. Work-Life Conflict and Gender Differences 

Work–family conflict is “a form of inter-role conflict in which the role pressures from the work and family domains are mutually incompatible in some respect” [22], (p. 77). In the current research, it is tackled as family-to-work or work-to-family interference, depending on the direction of the conflict being studied [59]. Considered the scope of this work, literature on the work-to-family conflict will be analyzed in this section. Interferences from work to nonwork life have been largely studied in organizational literature. Such conflict has been reported to reduce job and family satisfaction, as well as physical and psychological health in employees, in cross-sectional and longitudinal studies [60,61,62,63]. This is particularly true for non-collectivistic cultures, where work and nonwork roles are perceived as competing, and work and family do not contribute to one another [62,64]. Gender is another antecedent frequently tackled in literature on work to family conflict [62,65,66,67]. Overall, despite there is a general tendency to hypothesize that women are more interested by such interference than men, meta-analyses showed small or no effects with this regard [62,65]. Some authors claim that personal characteristics, as well as the gender balance within the participant samples, may have a role in influencing these findings [62]. Consistently with this heterogeneous view, studies on work-life conflict in school principals reported a higher need for female principals to juggle their work and private life when compared to male colleagues, as well as the choice by female principals to invest less on private life, to the benefit of work and career [29,30,68]. Thus, it would be interesting to further explore the role of gender when tackling work-life conflict in this profession.

### 1.3. Burnout and Gender Differences 

Previous studies about gender differences on burnout symptoms outlined that female workers were more likely to display higher level of exhaustion (as the core dimension of burnout syndrome), while male workers are more likely to report depersonalization [18,69]. This issue has been interpreted by two main perspectives [70]. Some authors interpreted gender differences in light of the strict link between burnout and depression symptoms (e.g., [35]). The latter is frequently associated to internalizing problems, which occur more likely in women than in men. A second and more interesting perspective, however, emphasizes the sociocultural dimensions involved in women’s professional choices and the psychological cost of their career development. Interestingly, some authors found that women are more likely to be burnt out and to experience anxious symptoms than men when they are occupied in male-typed professional roles [71,72,73]. Although, it is worth noticing that high levels of frustration and distress were found in men employed in female-type occupations [74]. Particularly interesting, in this regard, is the consideration that burnout and its corollarial symptoms are more likely to be observed in some professions than others (e.g., helping professions) [75,76,77,78], on one hand, and to be affected by gender stereotypes, on the other [79]. Past research on this matter has been particularly difficult due to several caveats, e.g., recruiting gender-balanced samples, controlling for socio-cultural variables, etc. The above-mentioned factors have contributed to a scarce literature on gender differences with mixed and inconsistent results. This is particularly true in the field of educational professions. In this regard, it would be beneficial for policy makers and practitioners to do a deeper investigation of gender differences by analyzing how and to what extent males and females differ in their work-related mental health dimensions. Furthermore, in a professional development perspective, it would be valuable to understand why and when work-related risk factors may increase their influence size and negatively impact on employees’ wellbeing in the case of both women and men. 

### 1.4. Theoretical Framework and Hypotheses

According to the Conservation of Resources (COR) theory [80,81], when people deal with stressful conditions, they either lose personal resources, perceive them as lost, or feel like they invested more than they are receiving back [82]. A main underpinning of this framework is that individuals constantly try to acquire, maintain and protect their personal resources, that are represented by objects, personal characteristics, conditions, or energies [80,82]. According to Hobfoll and colleagues, when a person experiences stress, and loses resources, his/her efforts are aimed at avoiding further losses, and conserve energy [80,82]. At the same time, it is known that this avoiding approach constitutes a loss of resources itself. This process constitutes a spiral of resource losses [80] and may regard all life contexts, regardless of the ones in which the resources got lost at first. Consistently, the COR theory was linked to several models explaining job-related stress and burnout, such as the Job Demands-Resources (JDR) model [83,84]. More specifically, JDR theory could be considered as an expansion of COR theory, thus showing the ultimate detrimental effects of resource loss cycles at work. According to Bakker, indeed, workers dealing with difficulties in organizations may end up depleting their resources, because their attempts to solve the problem, may aggravate the problem itself [85]. In this condition, when loss spirals become loss cycles, employees may encounter a total depletion of resources, thus leading to burnout [85]. In case of workplace bullying, the episodes may act as strains that ignite the spiral of resource loss. These episodes are usually connected to psychological and physical risk conditions, such as depression, deviant behaviors, anxiety and burnout [11,86,87,88]. 

Overall, the above mentioned literature informs about the chance that negative conditions at work, such as workplace bullying, act as direct sources of burnout [11,86,87,88]. For this reason, the first hypothesis of this work is the following:

**Hypothesis 1:**  *Workplace bullying is a burnout predictor*.

Frequently the workplace represents the first context of resources loss, and family the context in which resources are depleted because of the spiral. Several authors, indeed, acknowledged that work and family or private life are not distinct areas of one’s own life, recognizing a permeability between the two [16,58,64,89,90]. The COR theory [80,82], indeed, suggests that stressors occurring in the workplace may impact private life, too, and that this effect may be expressed as work-life conflict. Consistently, studies inform about the potential detrimental role of work-life conflict on burnout [91,92]. As described above, Kristensen [20] acknowledged, indeed, that burnout may interest people dealing with emotional exhaustion in several different life contexts, not only at the workplace. 

Therefore, the second hypothesis of this work is the following:

**Hypothesis 2:**  *Work-life conflict mediates the relationship between workplace bullying and burnout*.

Furthermore, as stated above, the literature has revealed the significant role of gender differences in the study of workplace bullying [39,40,41,42,43], work-life conflict [62,65,66,67], and burnout. Therefore, the third, fourth, and fifth hypotheses of this work are the following:

**Hypothesis 3a:**  *Gender moderates the relationship between workplace bullying and burnout*.

**Hypothesis 3b:**  *Gender moderates the relationship between workplace bullying and work-life conflict*.

**Hypothesis 3c:**  *Gender moderates the relationship between work-life conflict and burnout*.

Finally, to the best of our knowledge, no study addressed such relationships in the educational context, tackling in particular the school principal experience. This work is based on the experiences of these participants, considered their peculiar role at school. 

**Figure 1:**
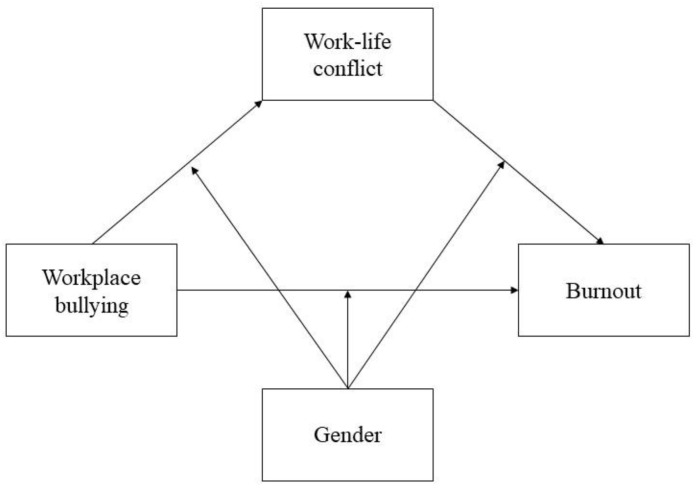
A conceptual model of the hypothesized moderated mediation.

The hypothesized model is shown in Figure 1. 

## 2. Materials and Methods 

### 2.1. Participants and Procedures

The participants were recruited through a collaboration with the Italian National Association of School Principals, which contacted 1798 principals in the whole Italian territory. Data were collected during Spring-Fall period of 2018. By the end of the data gathering, 1669 principals provided full, valid responses (participation rate = 92.8%). For the scope of this work, 860 participants were selected, so that the sample was balanced in terms of gender. More specifically, starting from the number of male participants in the study (*N* = 430), an equal number of females was randomly selected, by means of a IBM-SPSS command (namely Random sample of cases, in the Select cases function). Table 1 shows the demographic and work-related characteristics of the selected participants. 

Regarding bullying experiences, 54.8% of women and 49% of men reported to have been targets of bullying episodes at school, either through unpleasant teasing or through receiving violent threats, during the last 12 months. Despite almost half of bullied participants chose not to disclose the identity of the perpetrator, teachers and students were reported as the most frequent perpetrators for unpleasant teasing (51% of total bullying cases), and students’ parents for episodes related to violent threats (40% of total bullying cases). 

All subjects gave their informed consent for inclusion before they participated in the study. The study was conducted in accordance with the Declaration of Helsinki, and the protocol was approved by the Ethics Committee of LUMSA University (approval of the November 2019). 

### 2.2. Measures 

Data was collected through the Copenhagen Psychosocial Questionnaire II—COPSOQ II [93], adapted to the cultural and work setting of Italian principals. This tool measures several work-related domains, namely: demands at work, work organization and job contents, interpersonal relations and leadership, work-individual interface, values at the workplace, health and well-being, offensive behavior, with 43 scales in total. The high coverage of work features and experiences entitles COPSOQ to the assessment of job strains according to several burnout models (included JDR; [94]), while not being driven by one specific model [95]. Furthermore, it has been validated with regard to several professional categories, included school principals [96]. 

Considered the scope of this study, four scales were selected. The first two selected scales were unpleasant teasing (one item: “Have you been exposed to unpleasant teasing at your workplace during the last 12 months?”) and threats of violence (one item: “Have you been exposed to threats of violence at your workplace during the last 12 months?”). These scales were combined in a mean score, namely workplace bullying, which Cronbach’s alpha was = 0.58. According to COPSOQ guidelines, the unpleasant teasing and threats of violence scales were measured on a 5-point response scale, ranging from 0 to 100, and defined as: 100 = Yes, daily; 75 = Yes, weekly; 50 = Yes, monthly; 25 = Yes, a few times; 0 = No. 

The third scale selected for the scopes of this study is the burnout scale (four items, Sample item: “[In the last four weeks] How often have you been physically exhausted?”; Cronbach’s alpha = 0.94). According to COPSOQ guidelines, it was measured on a 5-point response scale, defined as: 100 = All the time; 75 = A large part of the time; 50 = Part of the time; 25 = A small part of the time; 0 = Not at all.

Finally, the fourth selected scale is the work-life conflict scale (four items, Sample item: “Do you feel that your work drains so much of your energy that it has a negative effect on your private life?”; Cronbach’s alpha = 0.86). According to COPSOQ guidelines, it was measured on a 4-point response scale, defined as: 100 = Yes, certainly; 75 = Yes, to a certain degree; 25 = Yes, but only very little; 0 = No, not at all. 

Finally, gender was recoded as a dummy variable (1 = male, 0 = female). 

### 2.3. Plan of Analysis

Descriptive statistics and correlational patterns were calculated to verify the associations between the variables. Furthermore, a moderated mediation analysis was performed [97], to verify the mediating effect of work-life conflict in the relationship between workplace bullying and burnout, and the moderating effect of gender in all the relationships verified by the mediating model. More specifically, the mediating effect was examined following the MacKinnon’s four-step procedure [98]. According to the first step the independent and dependent variables should be significantly associated. Thus, we verified the association between workplace bullying and burnout. In the second step, the independent variable and the mediator should be significantly associated. Thus, we verified the association between workplace bullying and work-life conflict. In the third step, the association between the mediator and the dependent variable should be significant, even when controlling for the effect of the independent variable. Thus, we verified the effect of work-life conflict on burnout, while controlling for the effect of workplace bullying. Finally, in the fourth step the indirect path between the independent and the dependent variable should be significant. Thus, we verified the indirect pathway between workplace bullying and burnout using the bias-corrected percentile bootstrap method, according to which the indirect path would be significant if the 95% bias-corrected confidence interval (CI) calculated with 5000 resamples excluded zero. Such analysis is provided by the macro PROCESS v.2.16 for IBM SPSS v.23 (IBM, Armonk, NY, USA) (Model 4) [99]. The same macro was used to verify the moderation of gender, too. More specifically, Model 59 of the macro PROCESS was used to test the moderated mediation model, namely whether gender would moderate the direct and the indirect effects of workplace bullying on burnout. As per the last path of the mediation model, if the 95% bias-corrected CI of the interaction did not include zero, the moderating effect of gender would be verified. Furthermore, bootstrap method was used to analyze the conditional effect at different values of gender (Male vs. Female). 

## 3. Results 

Table 2 shows the descriptive and correlation values for all the variables. As shown, all the variables showed significant associations to one another. 

Table 3 shows the results of the simple mediation model. As expected, workplace bullying has a significant effect on burnout (Hypothesis 1), and work-life conflict mediated this relationship (Hypothesis 2). More specifically, the total effect of workplace bullying on burnout was significant (*R*^2^ = 0.05, *F*_1,858_ = 30.98, *p* < 0.001). At the same time, the effects of workplace bullying on work-life conflict, of work-life conflict on burnout, and the direct effect of workplace bullying on burnout, resulted as significant. Furthermore, as the bootstrapped 95% confidence interval around the standardized indirect effect did not include zero, the indirect effect is significant. The results, indeed, show a partial mediating effect of work-life conflict in the relationship between work bullying and burnout (*R*^2^ = 0.38, *F*_2,857_ = 253.57, *p* < 0.001). 

Hypotheses 3a, 3b, and 3c tested, respectively, whether gender interacts with workplace bullying to predict burnout, with workplace bullying to predict work-life conflict, and with work-life conflict to predict burnout. As shown in Table 4, Hypotheses 3b and 3c were confirmed, while Hypothesis 3a was not confirmed. Overall, the model predicting work-life conflict that includes the interaction between gender and workplace bullying on work-life conflict is significant (*R*^2^ = 0.05, F_3,856_ = 20.80, *p* < 0.001), as well as the model including the interaction between gender and work-life conflict on burnout (*R*^2^ = 0.39, F_5,854_ = 110.85, *p* < 0.001). We studied the conditional indirect effect of workplace bullying on burnout (through work-life conflict) at different levels of gender: male (1) and female (0). Results (Table 4, Conditional indirect effects at different levels of Gender) showed that the conditional indirect effect was significant both for males and females. This datum confirms that gender does not show the three hypothesized moderating effects. 

As the mediation model (Table 3) showed a partial mediating effect, we studied the conditional direct effects of workplace bullying on burnout (without considering the role of work-life conflict) at different levels of gender (not shown). The conditional direct effect, namely the effect of gender when work-life conflict has an average score, was significant for female principals (Effect = 0.46, SE = 0.17, *t* = 2.66, *p* < 0.01), but not for male principals (Effect = 0.31, SE = 0.22, *t* = 1.43, *p* = 0.15). 

## 4. Discussion 

Overall, our findings showed that: 

Workplace bullying and burnout are associated, both with and without the perception of a concurring work-life conflict (thus confirming Hypotheses 1 and 2).

Gender does not moderate all the possible relationships among workplace bullying, work-life conflict and burnout. More specifically, while Hypotheses 3b (*Gender moderates the relationship between workplace bullying and work-life conflict*) and 3c (*Gender moderates the relationship between work-life conflict and burnout*) were confirmed, Hypothesis 3a (*Gender moderates the relationship between workplace bullying and burnout*) was not confirmed in our findings. At the same time, being female heightens the risk to perceive work-life conflict in general, as well as being burnt out, when bullied, with and without the presence of work-life conflict; being male heightens the risk to perceive work-life conflict when bullied. 

### 4.1. Permeability between Work and Private Life

Regarding the first point of our results, namely the association between bullying and burnout, with and without the mediating effect of work-life conflict, our data confirms previous literature about the detrimental effects of workplace bullying on employees’ and managers’ health [11]. Furthermore, findings confirmed previous data about the permeability between work and family life contexts [16,58,64,89,90]. 

At the same time, this work extends earlier findings to a less studied occupation, namely school principals. 

As claimed by several authors, the increasing, variegated and potentially conflicting job demands that school principals are required to fulfill may act as strains, that, in turn, could endanger their occupational and personal health [3,52,100,101,102]. School principals’ position, indeed, is at the crossroad between a helping profession and a public administration role [24,25,26,27,28,103]. This duality influences the way principals perceive their work experiences, likely influencing their professional and personal identity [29,104]. 

Previous studies, indeed, underline the centrality of relationships at work for school administrators. For example, Leithwood and colleagues showed that one of the main leadership practices at school is developing people [105,106]. According to Ishimaru, indeed, to develop student achievement and growth, educators, parents, and communities need to relate and collaborate one another, and principals are called to foster and promote these links [107]. Despite such role, principals are frequently described as socially isolated within schools. According to Bauer, their professional and social isolation is because they are the only administrators in charge of school outcomes and decision making [51]. Consistently, Howard and Mallory stated that isolation, together with the stress related to the job, are frequently connected to role conflict, ambiguity and overload [53]. Finally, principals may suffer from physical and emotional exhaustion because of the lack of social proximity at work [54]. By combining previous literature with our findings, it seems that school principals may be at risk for workplace bullying and burnout, because of the nature of their job. These studies, indeed, suggest that school principals may be specifically at risk for the Hobfoll’s spiral of resource losses [80], as the energy deterioration seems to begin in the workplace, because of the job demands typical of their position. This depletion, in turn, may bring to personal burnout. To the best of our knowledge, current literature debates about how and when such mechanisms may influence private life only when addressing the experience of female school principals, alone or as opposed to male principals’ one [30,108,109]. Despite this, our findings suggest that family needs and demands may influence the feelings of exhaustion of all school principals, independently from their gender. Further research is needed to better tackle male principals’ caring experience and work-life balance. 

### 4.2. Gender Differences in Dealing with Workplace Bullying

Within the general relationships described in the previous section, the moderated mediation model disclosed some specific effects related to principals’ gender. More specifically, a double risk emerged for female principals, regarding the likelihood to perceive higher work-life conflict than male colleagues, as well as higher likelihood to be burnt out, when bullied, with and without the presence of work-life conflict. 

These results contribute to the debate about the role of gender in influencing the risk for burnout [18,69,70] and work-life conflict [62,65,66,67], by verifying it in a specific job role. 

The role of gender, indeed, is specifically interesting when addressing principals work experience, as previous works suggest that gender may lead to significant differences in how principals perceive themselves and their role at school [30,68,110,111,112]. This is even more relevant when considering the rates of female principal as they are reported in international surveys. According to OCSE TALIS 2018 data, whose sampling strategy is a systematic random sampling with probability proportional to size, in 20 out of 47 countries involved in the survey, female principals covered more than the 50% of the sampled schools. This finding suggests an overall balanced distribution of gender within this working population. At the same time, when looking more specifically to gender distribution in the country of interest of this work, namely Italy, TALIS 2018 data show that female principals represented almost the 70% of the sample [113]. Considering that in Italy school principals always have a previous career as teachers, this datum is consistent with findings on the prevalence of females within the overall Italian teacher population [113]. Thus, it is likely that female principals constitute the main part of the school leaders’ workforce in the national context of this study. 

Building on the literature on the caring role of school principals, some studies underlined the higher tendency of female principals in representing their role as mostly relationship-based, and, thus, in opting for a transformational, caring leadership style [30,109]. This approach to school management, according to Eckman’ interviewees, is not merely aimed at fostering a positive relational climate, but above all at acting as role models for other women in the school [30]. According to Smith, such vision of school management, despite being neglected by bureaucratic-based interpretations of leadership at school, are beneficial to the school community, above all regarding the management of school-family relationships [109]. At the same time, Grissom and colleagues studied the effects of having female principals within the school staff, shedding an interesting light on the positive representation of female principals at school [108]. Comparing the degree of satisfaction and turnover in female vs. male teachers working in schools administered by female vs. male principals, the authors showed that male teachers were less satisfied and more likely to turn over when their principal was a woman. Overall, these studies suggest a contradicting representation of female leadership at school. It is likely that this condition, together with the isolation and the role conflict and overload described above, may heighten the risk of female principals to be targeted by workplace bullying. Considered the people-oriented leadership style suggested by previous works for female principals, it is likely that these relationship-based conflicts may heighten the risk for burnout, too, in case of bullying from families or teachers. 

Findings displayed a risk for male principals, too, regarding the higher likelihood to perceive work-life conflict when bullied, if compared to female colleagues. Previous studies on workers from other organizations showed that men are interested by more severe affective symptoms, such as anxiety and feeling of hopelessness, when targeted as workplace bullying victims [114]. Previous studies suggested that the higher impact, when compared to female experience, may be due to male self-representations as workers [115]. Previous scholars [116], indeed, showed that men and women represent the experience of victimization from different points of view. While women are more likely to describe the bullying episode as originated by a group dynamic and the victim as being a scapegoat, men consider victimization as the expression of personal failure, lack of competence, and weakness. According to some authors, such representations may be due to the feeling of being a gender-atypical condition, thus hypothesizing a role of gender stereotypes [117,118]. At the same time, Attell and colleagues extend such representations to the family context, underlining that the provision of economic support is a key task that men attribute to themselves as part of their contribution to the family life [114]. Being bullied may question such a role, as it is related to a low sense of safety within the workplace, as well as to low self-efficacy as a professional [8,10]. 

### 4.3. Practical Implications

Our findings offer important hints for addressing principals and headmasters’ stress. Our findings showed several links to the literature on the caring vs. administrative role of principals, above all by explaining the role of female principals as mostly care- and people-oriented. This might be explained in the light of the fact that, regardless of gender, almost all Italian principals, included the participants, are teachers with a long-term experience, who upgraded to the principal role after passing a selective, national exam. Therefore, it might be hypothesized that principals tend to reverse or—better say—re-use the strategies already learned for managing the dynamics of their classes in their new tasks [119,120]. If these skills might undoubtedly be useful and effective [121], on the other hand might not be exhaustive of the competences and abilities required by the new role. In other words, we cannot assume that a teacher might be sufficiently equipped to cope with his/her new managerial tasks, no matter how positive and long is her/his teaching experience. As shown by previous studies [100], independently from gender, principals might well lack of the knowledge and skills required to handle the school dynamics from a new, different perspective. In fact, teachers deal with children and young people as well as colleagues and families, while principals deal with students, teachers, families and more stakeholders, such as non-teaching staff and as external stakeholders such the Ministry of Education, suppliers, organizations involved in partnership with their schools. Examples of these skills are: mediation, negotiation, strategic thinking, long-term planning, team building, and conflict management. Thus, it would be interesting to broaden the present findings in future studies by taking into account these skills and its potential moderating role. Furthermore, training principals to develop the abovementioned abilities might offer a substantial help to effectively address and prevent the highly stressing situation they have to face. More in general, the literature on the role played by managers in sustaining their employees’ performance and well-being shows the relevance of addressing the training of principals not only about the norms and the rules of the school administration, but also about the psychosocial and managerial practice and methods. 

### 4.4. Limitations 

This study is not without limitations. Firstly, a longitudinal or mixed-methods approach would have given this work more depth. In the first case, by tackling more directly the risk for displaying personal burnout as originated by workplace bullying; in the second, by taking into account principals’ representations and motivations related to workplace bullying and relative coping strategies, in and out of school. At the same time, considering the current mixed results on the role of gender in these topics, further research could enrich our understanding by adopting qualitative methods to tackle school principals’ experience. Secondly, recent research showed a distinction between workplace bullying and workplace interpersonal conflict [49]. The lack of measures that could allow the researchers to differentiate between the two conditions does not allow to define whether some of the behaviors reported by the participants rely more on interpersonal conflict that on actual bullying experiences. Furthermore, since only a small part of psychosocial workplace factors was taken into account in the present study, practical implications and improvement suggestions should be limited to these factors. Another consideration regards inverse mediating models: we did not test a model in which, for example, burnout mediates the relationship between workplace bullying and work-life conflict. A similar model was tested by Raja and colleagues [6]. At the same time, considering the cross-sectional nature of this study (opposed to Raja and colleagues’ longitudinal work), as well as the focus on gender differences, the authors preferred not to test an inverse model. Furthermore, one of our scales has a Cronbach’s alpha value slightly lower than 0.60 (which is usually considered as an acceptable cut-off). While it may be due to the specific sample, or to the fact that, on average, participants did not experience frequent bullying behaviors at victims, further researches could help defining whether such scales from COPSOQ could provide an overall, reliable, measure of bullying experiences at work or not. Moreover, considering the specific national context in which the study was conducted, the generalization of results to other countries should be taken with caution. European studies, for example, showed a heterogeneous distribution of exposure to adverse social behaviors at work among countries. For example, in the most recent European Survey on Working Conditions, the percentages of women reporting such behaviors ranged from 3 to 29 percent [122]. Thus, practitioners and policy makers would benefit from confronting models on the detrimental effect of workplace bullying among different countries. Finally, it would be interesting to interpret data on workplace bullying towards school principals by gathering more data on school contexts and teachers, students, and families’ representations of their school leaders. The lack of such information did not allow the authors to deepen the interpretation of the findings. 

## 5. Conclusions

Overall, this work shed a new light on the interplay between workplace bullying, work-life conflict and burnout, above all with regard to the role of the target’s gender. More specifically, we found that, as could be expected building on previous literature, workplace bullying and burnout are associated, both with and without the perception of a concurring work-life conflict. At the same time, with regard to the role of gender, we found that it does not moderate all the possible relationships among workplace bullying, work-life conflict and burnout. More specifically, being female heightens the risk to perceive work-life conflict in general, as well as being burnt out, when bullied, with and without the presence of work-life conflict; being male heightens the risk to perceive work-life conflict when bullied. These findings may inform about the need of training programs for principals, aimed at strengthening their professional identity, and foster soft skills such as mediation, negotiation, strategic thinking, long-term planning, team building, and conflict management.

## Figures and Tables

**Table 1:** Demographic information table of the participants.

Variable	Total (*n* = 860)		Male (*n* = 430)		Female (*n* = 430)		Gender Differences	
	*M*	*SD*	*M*	*SD*	*M*	*SD*	*t(df)*	*p*
Age	56.13	5.53	56.54	5.80	55.7	5.23	2.189 (856)	0.03
Years of experience as school workers	29.46	8.46	29.89	8.40	29.02	8.50	−1.388 (858)	0.17
	**Frequencies (%)**		**Frequencies (%)**		**Frequencies (%)**		***Cramer’s V***	***p***
Type of school administered ^1^							0.16	<0.01
Primary/middle schools	333 (38.7)		200 (46.5)		133 (30.9)			
Secondary schools	411 (47.8)		175 (40.7)		236 (54.9)			
Primary/middle/secondary schools	73 (8.5)		35 (8.1)		38 (8.8)			

Note. *M* = Mean; *SD* = Standard deviation; *t* = t-test; *df* = degree of freedom; *p*= significance. ^1^ = 5% of participants did not provide such information.

**Table 2:** Descriptive statistics and correlation values.

Variables	Descriptive Statistics		Correlations			
	Mean	Standard Deviation	Bullying	Burnout	Work-Life Conflict	Gender
Bullying	6.56	10.18	-	0.215 **	0.187 **	−0.075 *
Burnout	60.79	23.14		-	0.606 **	−0.135 **
Work-life Conflict	76.99	22.34			-	−0.090 **
Gender						-

Note. * = *p* < 0.05, ** = *p* < 0.01.

**Table 3:** Regression results for simple mediation.

Variable	*B*	*SE*	*t*	*p*
**Direct and total effects**				
Workplace bullying → Burnout	0.82	0.15	5.57	<0.001
Workplace bullying → Work-life conflict	0.69	0.09	7.42	<0.001
Work-life conflict → Burnout (controlling for Workplace bullying)	0.61	0.03	21.21	<0.001
Workplace bullying → Burnout (controlling for Work-life conflict)	0.40	0.14	2.90	<0.01
Sobel test	***Value***	***SE***	***z***	***p***
	0.42	0.06	7.00	<0.001
Bootstrap results for indirect effect	***Bootstrapped indirect effect***	***Boot SE***	***Boot*** ***LLCI***	***Boot*** ***ULCI***
	0.42	0.06	0.301	0.547

Note. Standardized regression coefficients are reported. Listwise N = 860. Bootstrap sample size: 5000. LLCI: lower-level bootstrap confidence interval; ULCI: upper-level bootstrap confidence interval.

**Table 4:** Results of Moderated Mediation Analyses.

Predictor	Burnout				Work-Life Conflict			
	β	SE	LLCI	ULCI	β	SE	LLCI	ULCI
Workplace bullying	0.39 **	0.14	0.11	0.66	0.69 ***	0.09	0.51	0.87
Gender	−3.46 **	1.27	−5.95	−0.98	−3.39 *	1.49	−6.32	−0.46
Work-life conflict	0.61 ***	0.03	0.55	0.67				
Workplace bullying × Gender	−0.15	0.28	−0.70	0.40	0.45 *	0.19	0.09	0.82
Work-life conflict × Gender	−0.16 **	0.06	−0.28	−0.05				
R^2^	0.39 ***				0.045 ***			
Indirect effects								
Conditional indirect effects at different levels of Gender	Bootstrapped indirect effect		Boot SE		Boot LLCI		Boot ULCI	
Female	0.32		0.09		0.14		0.50	
Male	0.49		0.08		0.33		0.66	

Note. * = *p* < 0.05, ** = *p* < 0.01, *** = *p* < 0.001. Bootstrap sample size: 5000; LLCI: lower-level bootstrap confidence interval; ULCI: upper-level bootstrap confidence interval; Listwise valid N = 846.
